# Small Bowel Intussusception due to Nasojejunal Tube Placement in Neonates: A Case Report

**DOI:** 10.70352/scrj.cr.25-0228

**Published:** 2025-07-19

**Authors:** Kazuki Shirane, Kyoko Mochizuki, Azusa Sugita, Satoshi Tanaka, Rento Morishima, Takafumi Kondo, Yoshinori Inagaki, Hidehito Usui, Norihiko Kitagawa, Katsuaki Toyoshima, Masato Shinkai

**Affiliations:** 1 Department of Surgery, Kanagawa Children’s Medical Center, Yokohama, Kanagawa, Japan; 2 Department of Neonatology, Kanagawa Children’s Medical Center, Yokohama, Kanagawa, Japan

**Keywords:** small bowel intussusception, nasojejunal tube, neonate, infant, ileal atresia

## Abstract

**INTRODUCTION:**

Nasojejunal tube placement is a rare cause of small bowel intussusception. It is usually treated with tube removal, but a few cases require surgical reduction. We report a case of small bowel intussusception due to a nasojejunal tube shortly after surgery for ileal atresia, in which surgical reduction was required despite removal of the tube.

**CASE PRESENTATION:**

A female infant underwent intestinal anastomosis for type III-A ileal atresia on the day of birth. A nasojejunal tube was placed for abdominal decompression until the temporary anastomotic passage obstruction improved. Bowel dilatation was successfully resolved; however, bilious gastric residuals suddenly increased again on postoperative day (POD) 11. Sonography revealed small bowel intussusception around the nasojejunal tube. As spontaneous reduction did not occur after tube removal, surgical reduction using the Hutchinson technique was performed on POD 20, including the release of adhesions between the intussusceptum and intussuscipiens of the jejunal intussusception. The patient experienced an uneventful course after surgical reduction.

**CONCLUSIONS:**

Sonography should be performed to screen for small bowel intussusception in patients presenting with bilious vomiting during nasojejunal tube placement. The prompt removal of the tube following a diagnosis of small bowel intussusception (SBI) is essential to prevent adverse events, such as adhesions between the intussuscepted bowel loops. If intussusception does not resolve shortly after tube removal, surgical intervention is indicated.

## Abbreviations

GJ
: gastrojejunal
NG
: nasogastric
NJ
: nasojejunal
SBI
: small bowel intussusception

## INTRODUCTION

SBI in children frequently occurs idiopathically; however, it is sometimes associated with a pathogenic lead point.^1,2)^ Although these 2 causes of SBI have been well documented, other rare mechanisms exist. Several reports have highlighted SBI caused by feeding tubes placed in the jejunum.^3–10)^ In the pediatric population, GJ tube placement has been recognized as a potential cause of SBI.^4–7,9,10)^ Recent reports suggest that SBI due to a GJ tube occurs in 1.3%–3.7% of patients with GJ tube placement.^4,9)^ In contrast, SBI caused by an NJ tube is rare, with very few documented cases.^3,11,12)^ We herein report a case of SBI due to an NJ tube following intestinal anastomosis in a neonate with ileal atresia. This case report provides key information regarding the diagnosis and treatment of this rare condition.

## CASE PRESENTATION

**Fig. 1:**
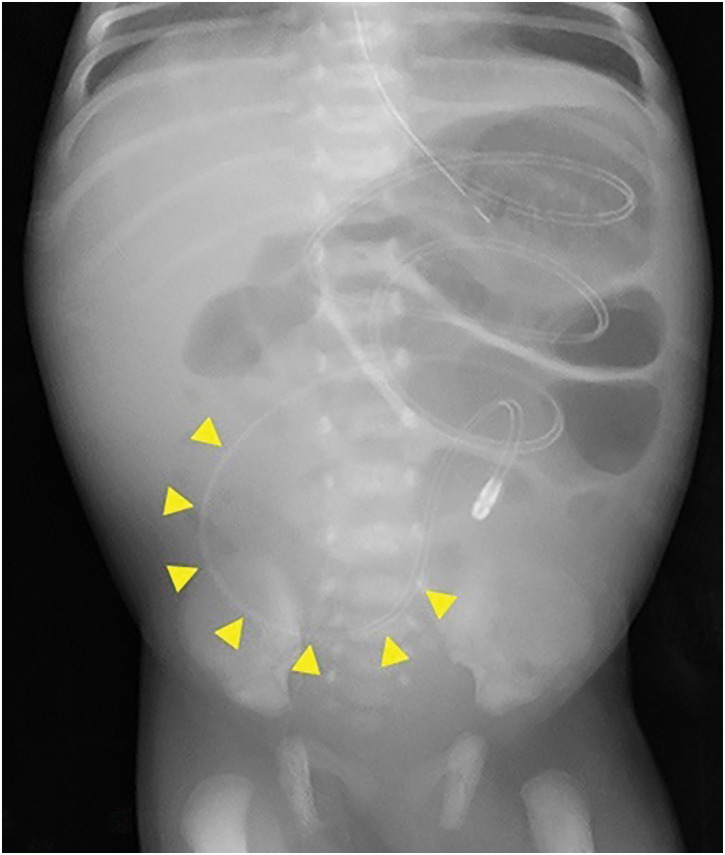
Abdominal radiography following the nasojejunal tube placement. There was no bowel dilatation proximal to the nasojejunal tube tip (arrowheads). However, radiography showed localized small bowel dilatation proximal to the left upper abdomen.

**Fig. 2:**
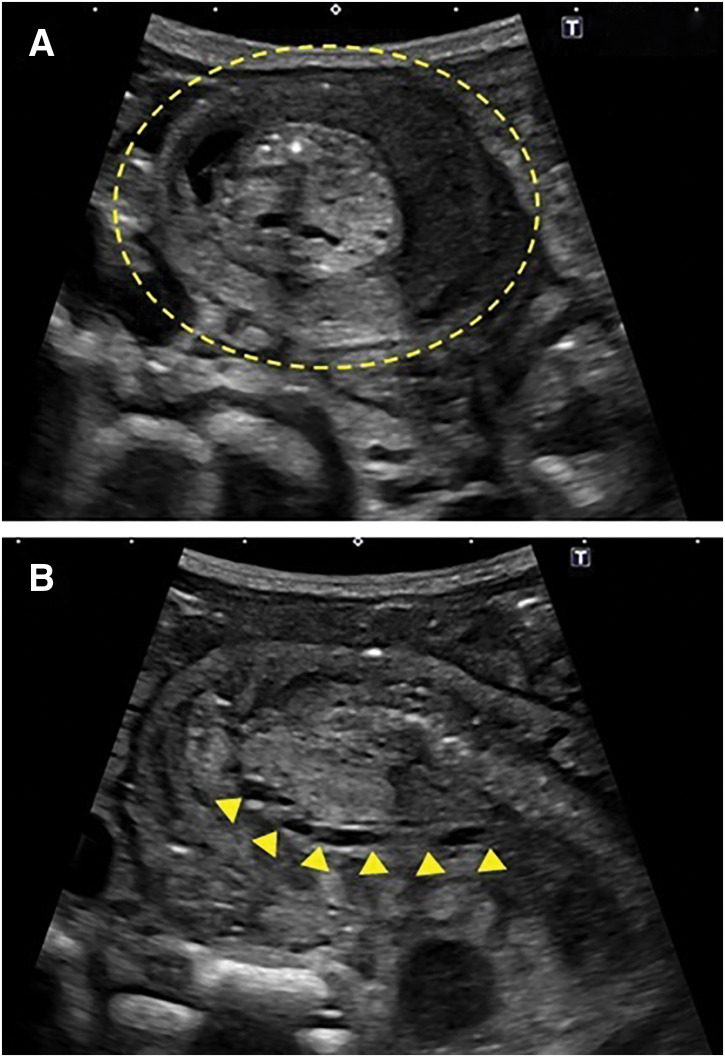
Sonography showing the small bowel intussusception. (**A**) Sonography showing the target sign in the right lower abdomen (dashed circle). (**B**) The nasojejunal tube passes through the intussuscepted bowel loops (arrowheads).

A female infant was born at 37 weeks of gestation with a birth weight of 2753 g. She underwent intestinal anastomosis for type III-A ileal atresia on the day of birth. A few days after surgery, she developed progressive abdominal distension despite gastric decompression through an NG tube. A 5-Fr NJ tube with a spindle (Cardinal Health, Tokyo, Japan) was placed in the jejunum for small bowel decompression. Enteral feeding was gradually initiated through the NG tube on POD 6, as the anastomotic passage improved. The NJ tube was left in place and kept open to monitor changes in drainage volume. On POD 11, bilious gastric drainage through the NG tube suddenly increased. Interestingly, the patient had regular bowel movements, sufficient stool output, minimal output from the NJ tube, and no abdominal distension. A radiograph showed no bowel dilatation around the NJ tube tip but revealed further proximal bowel dilatation localized in the left upper abdomen (**Fig. 1**). Subsequent sonography revealed SBI along the NJ tube on POD 12 (**Fig. 2A** and **2B**).

**Fig. 3:**
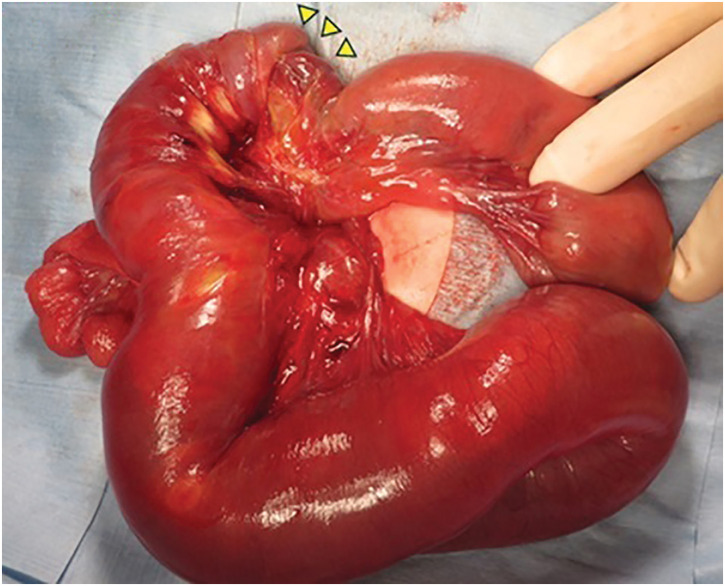
Jejunal intussusception with adhesions was revealed at laparotomy. The proximal bowel was invaginated into the distal bowel, indicating antegrade intussusception (arrowheads). Adhesions were observed between the inner and outer segments of the intussuscepted bowel loops.

As it is still under discussion whether the increased gastric drainage was caused by SBI or anastomotic passage obstruction, the NJ tube was left in place. Even after the onset of SBI, reinfusion of gastric drainage and nutritional administration were successfully performed through the NJ tube. This situation helped rule out the recurrence of anastomotic passage obstruction. The NJ tube was removed on POD 17. However, laparotomy was required on POD 20 because of failure of spontaneous reduction. Antegrade jejunojejunal intussusception was identified 20 cm distal to the ligament of Treitz, with adhesions between the intussusceptum and intussuscipiens (**Fig. 3**). The SBI was clearly distant from the anastomotic site, located 85 cm distal to the ligament of Treitz. Manual reduction using the Hutchinson technique was performed after the release of the adhesions. She had an uneventful course following the reduction of SBI and was discharged on the 46th day after anastomosis for ileal atresia.

## DISCUSSION

SBI caused by NJ tube placement is a rare condition that generally has a favorable prognosis because it typically resolves spontaneously after tube removal.^3,7,10)^ However, we demonstrated that a delay in tube removal may cause adhesions of the invaginated bowel loops that require surgical intervention. Moreover, this study highlights the diagnostic importance of focusing not only on the symptoms of bilious vomiting but also on the amount of drainage from the NJ tube tip to facilitate the early detection of SBI.

SBI associated with NJ tubes has been reported less frequently compared to SBI associated with GJ tubes. To explain this difference, we focused on the tube size. GJ tubes are generally larger in diameter than NJ tubes. For example, 16-Fr GJ tubes are commonly used,^4,7)^ whereas NJ tubes are typically between 6 and 10 Fr.^13)^ Disproportion between tube size and intestinal diameter has been identified as a potential risk factor for SBI.^3)^ Therefore, the relatively large size of GJ tubes may be associated with a higher risk of SBI compared to NJ tubes. Nevertheless, our case suggests that even a 5-Fr NJ tube may be relatively large and carry a potential risk of SBI in neonates.

Vomiting, which is often bilious, is the most common symptom of SBI caused by GJ tube placement.^10)^ However, it is not diagnostically specific because there are several other potential causes, such as anastomotic site obstruction and adhesive small bowel obstruction. These conditions are more common in patients with a recent history of surgery. In our case, enteral nutrition was successfully provided through the jejunal tube, and there was sufficient stool output. This was not consistent with the re-obstruction at the site of anastomosis. This situation could only be explained by an obstruction between the duodenum and the tip of the NJ tube. We believe that these findings may be indicators of NJ and GJ tube-related SBI.

Most cases of SBI caused by GJ or NJ tubes resolve spontaneously after tube removal.^3,7,10)^ However, surgical intervention is sometimes required with either type of tube.^6,8,10,12)^ Our patient failed to achieve spontaneous reduction due to strong adhesions between the intussusceptum and intussuscipiens. Since the intussusception was clearly distant from the anastomotic site for ileal atresia, the adhesions were likely due to chronic inflammation associated with prolonged intussusception. Because spontaneous reduction without tube removal has not been reported, immediate tube removal is essential. On the other hand, the 3-day interval between NJ tube removal and surgical reduction in this case may have been too long. According to a previous report, spontaneous reduction was confirmed by an ultrasonographic follow-up within 24 h in most SBI cases that was resolved without surgical intervention.^2)^ Therefore, the switch to surgical reduction should be decided within 1–2 days after tube removal.

We hope that this case report will aid in the early detection and treatment of intussusception caused by NJ tubes in the future.

## CONCLUSIONS

SBI caused by NJ tube placement is rare, but may require surgical intervention. This case highlights that bilious vomiting without increased drainage from the tube tip is an important symptom that may contribute to the early detection of SBI. Our case also indicates that delayed removal may result in adhesions of the invaginated bowel loop and lead to poor outcomes. Therefore, the tube should be removed immediately after the detection of SBI. If spontaneous reduction does not occur soon after tube removal, prompt surgical intervention should be performed.

## ACKNOWLEDGMENTS

We would like to thank Japan Medical Communication for the English language editing.

## DECLARATIONS

### Funding

No financial support was provided.

### Authors’ contributions

KS and KM performed the surgeries.

KS drafted the original manuscript.

KM revised the original manuscript.

AS and YI provided perioperative care to the patient and contributed to the clinical course described in the manuscript.

ST, RM, TK, HU, NK, KT, and MS critically reviewed the manuscript.

All the authors approved the final version of the manuscript.

### Availability of data and materials

The datasets for this case study can be obtained from the corresponding author upon a reasonable request.

### Ethics approval and consent to participate

This work did not require ethical considerations or approval. Informed consent to participate in this study was obtained from the patient’s parents.

### Consent for publication

Informed consent for publication of this case report was obtained from the patient’s parents.

### Competing interests

The authors declare no conflicts of interest in association with the present study.
